# Supplementary material for: Determinants of mortality among preterm neonates admitted at Wolaita Sodo University Comprehensive Specialized Hospital, southern Ethiopia: An unmatched case-control study
Source: PLoS One. 2024 Dec 2;19(12):e0314632. doi: 10.1371/journal.pone.0314632 (PMC11611175; doi:10.1371/journal.pone.0314632)
Supplement: Attachment — Submitted filename: Response to Reviewers 2nd.docx [file pone.0314632.s002.docx]

As a first step, we express our gratitude to the academic editor for giving us enough time to rewrite and answer all of the reviewers' issues. The reviewer's remarks were carefully considered, and any necessary revisions or changes were made. For comparison, we addressed each concern with a response to the reviewer's comment. We hope that a much better article will be sent to the expert reviewers and academic editor. Many thanks for that!

**Journal Requirements**: “Please review your reference list to ensure that it is complete and correct. If you have cited papers that have been retracted, please include the rationale for doing so in the manuscript text, or remove these references and replace them with relevant current references. Any changes to the reference list should be mentioned in the rebuttal letter that accompanies your revised manuscript. If you need to cite a retracted article, indicate the article’s retracted status in the References list and also include a citation and full reference for the retraction notice.”

Dear Academic editor, Based on your suggestions regarding journal requirements, we replaced some retracted references with similar ones, cited online sources for some of the guidelines we used, and replaced references from the old edition of the book (Nelson text book 21st ed) with the current edition (Nelson text book 22st ed). Please find attached a marked-up copy in the reference section of the main manuscript. Regards!!

**Authors response for Reviewer 1**

1.“Line #111, in addition to health institution, better to add other infrastructure (i.e. educational institution, etc) in the town.”

Response: Dear Reviewer, per you comment, we added it. There are roughly 156 educational institutions in Sodo Town overall (63 public and 93 private). In addition to one university, there are five colleges, five secondary and preparatory schools, seven international hotels, and seven road networks that connect the various Woredas in the area and the surrounding areas. Thanks!

2. “Line # 124, better if you explain/ add the mix of health professional with respective to specialty, sub-specialty working in NICU.”

**Response**: Dear Reviewer, We have added it. As explained here: The pediatrics and child health department employs nine pediatricians, twenty-three residents, four general practitioners, and six to eight medical interns who rotate monthly between the NICU and other departmental units. Of these, one pediatrician, four residents, one general practitioner, and two to three medical interns work in the NICU each month and 17 trained nurses with patient to nurse ratio of six to one respectively.” Thanks!

3. “Line # 147 ' incomplete chart' is indirectly excluded. no need of write again as excluded.”

**Response**: Dear reviewer, we omitted it in main manuscript: Recorded data of preterm babies with no clear end outcome ~~or incomplete chart~~ or referrals or those who were early self-discharged against medical advice and neonates with lethal congenital malformations were excluded. Thanks!
4. “Line # 182-183, from where you recruit the data collectors and supervisors; what is your criteria to select them as data collectors or supervisor?”

**Response**: Dear reviewer, In order to address this issue, we attempted to rewrite our data quality assurance section in response to your query. The data collectors were nurses and medical interns who worked part-time in the NICU and were well-versed in medical terminology and documentation: In addition to one public health expert supervisor, three BSc nursing professionals and two medical interns who were working in the NICU, had a solid grasp of medical terminology, and were acquainted with NICU documentation on patient charts and registries were assigned as data collectors on a part-time basis. Thanks!!

5. “For me, Table 1 and 2 are not important. Better if you use graph for either of the two.”

**Response:** Dear reviewer, We reduced the number of tables by using graphs for some of the variables and combining the tables into a single table based on your feedback; however, it became difficult for us to display all of the variables (roughly 15-20) in a single table on graphs, so we combined tables 1 and 2 into a single table and used graphs for some of the variables, as shown in the main manuscript figure part.**Thanks**!

**Authors response for Reviewer “Reviewer #3:**

1. “The purpose of the research should be clearly stated in the abstract. The difference between this study and previous studies should be mentioned in the introduction (creativity and innovation)...”

**Response:** dear reviewer, based on your suggestion we tried to modify abstract in order to clarify the purpose of this study: as explained here, Although the general newborn mortality rate in the research area was 27 per 1000 neonates, but the preterm neonatal mortality rate and determinants were not previously well recognized. This study aimed to identify the Determinants of preterm neonatal mortality admitted to WSUCSH.

**2) “**The difference between this study and previous studies should be mentioned in the introduction (creativity and innovation)...”. Dear reviewer, We attempted to include what is novel in this study in comparison to earlier studies in introduction part based on your advice:- Additionally, as mentioned in the methods section, the research environment is different from other similar study locations in terms of the availability of medical equipment and medical specialists as well as the inclusion of other factors that may contribute to mortality (e.g., pregnancy care: induction of delivery, medical disorders: thyroid disorder; newborn characteristics: weight to GA and apnea of prematurity, length of hospital stay (LOS) were taken into consideration; LOS was one of the significant factors according to this study in addition to others). **Thanks**!
